# Alternative Strategies in Response to Saline Stress in Two Varieties of *Portulaca oleracea* (Purslane)

**DOI:** 10.1371/journal.pone.0138723

**Published:** 2015-09-23

**Authors:** Kristina R. Mulry, Bryan A. Hanson, Dana A. Dudle

**Affiliations:** 1 Dept. of Chemistry & Biochemistry, DePauw University, Greencastle, Indiana, United States of America; 2 Dept. of Biology, DePauw University, Greencastle, Indiana, United States of America; Chinese Academy of Sciences, CHINA

## Abstract

Purslane (*Portulaca oleracea*) is a globally-distributed plant with a long history of use in folk medicine and cooking. We have developed purslane as a model system for exploring plant responses to stress. We exposed two varieties of purslane to saline stress with the objective of identifying differences between the varieties in the plasticity of morphological and physiological traits. The varieties responded to saline stress with significantly different changes in the measured traits, which included *inter alia* biomass, flower counts, proline concentrations and betalain pigment concentrations. The alternative responses of the two varieties consisted of complex, simultaneous changes in multiple traits. In particular, we observed that while both varieties increased production of betalain pigments and proline under saline stress, one variety invested more in betalain pigments while the other invested more in proline. Proline and betalain pigments undoubtedly play multiple roles in plant tissues, but in this case their role as antioxidants deployed to ameliorate saline stress appears to be important. Taken holistically, our results suggest that the two varieties employ different strategies in allocating resources to cope with saline stress. This conclusion establishes purslane as a suitable model system for the study of saline stress and the molecular basis for differential responses.

## Introduction

Purslane (*Portulaca oleracea*) is succulent, robust medicinal plant, distributed in temperate and tropical regions world-wide. It thrives not only in gardens and cultivated fields, but in waste spaces and sidewalk cracks. It has been used extensively in folk medicine since antiquity, and also as a nutritious pot herb and salad ingredient. [1–3]

Purslane has been investigated extensively for bioactive compounds. [4] The most noteworthy are the *ω*-3 fatty acids, and in particular, *α*-linolenic acid (ALA). This class of compounds and their metabolites have been identified as inhibitors of tumorigenesis [5] and promoters of good cardiovascular health. [6] Among terrestrial sources, purslane has the highest amount of ALA, with approximately 4 mg ALA per gram of fresh plant. [7] Purslane is also rich in antioxidant compounds and it activates enzymes that quench oxidants. [8–10] Like most members of the order Caryophylallales, purslane synthesizes betalain pigments rather than anthocyanins. [11, 12] These pigments are antioxidants and of great interest as natural food colorants and nutritional supplements, [13] and have been shown to protect low density lipoprotein particles. [14] Polysaccharides are abundant as well, and exhibit antiviral, antidiabetic and antitumor activities. [15–17] The seeds [18] and aqueous extracts [19, 20] have been found to be useful in the control of diabetes and to ameliorate some side effects of the disease. Finally, purslane extracts have shown neurological effects worthy of further investigation. [21, 22]

The high level of ALA has led researchers to investigate the conditions necessary to optimize the production of fatty acids. The influence of nitrogen source [23], stage of harvest [24] and light [25] have been reported. Purslane has also been studied for the remediation of soils and industrial effluents polluted with metals [26], salt [27] and endocrine disruptors like bisphenol A. [28] An interesting characteristic of the genus *Portulaca* is that it contains the only known example of a C-4 plant which can switch to crassulacean acid metabolism under drought stress, and *P. oleracea* in particular does not have photosynthetic stems. [11, 29, 30]

We have identified purslane as a suitable model system for exploring plant responses to stress, particularly saline stress. Recent modeling of anthropogenic climate change predicts greater extremes in temperature and rainfall, with a net increase in global temperature and a decrease in rainfall. These changes are not expected to be evenly distributed, and it is anticipated that semi-arid regions will expand significantly. [31] Higher temperatures in agricultural regions will lead to increased irrigation, which in turn will increase soil salinity in an unpredictable pattern across the range of widespread species such as purslane. At the same time, evidence is mounting that plant species ranges and community assemblages are already responding to climate change in complex ways. [32, 33]

Because plants are sessile, individuals that exhibit phenotypic plasticity in response to environmental stressors may buffer the effects of unpredictably harsh environments resulting from climate change. Such plasticity may allow some individuals to colonize hostile sites and reproduce despite saline conditions. [34] Adaptive plasticity in functional traits such as leaf mass and presence and concentration of secondary metabolites has been identified as a key factor in the effectiveness of plants’ response to unpredictable salinity within a generation. [35]

Over longer time frames, natural selection should favor plants with greater ability to cope with saline soils and other stressors as the climate changes more permanently. These genotypes must have functionally integrated biochemical phenotypes that improve their fitness and survival. [36] At the scale of plant populations and communities, extirpation is a real possibility if species cannot adapt quickly, disperse into suitable habitats or find refugia. Thus, climate change not only threatens crops and the food supply, but also biodiversity and the accompanying ecosystem services.

We report here our investigations into the responses of two varieties of purslane to saline stress. In particular, we investigated whether both varieties exhibit the same level of plasticity in response to saline stress, and whether functional traits such as biomass, flower production, and concentration of secondary metabolites respond to saline stress in a coordinated fashion, using ecological and chemical methods. Because purslane is widespread and synthesizes a wide array of bioactive compounds that are likely associated with adaptation to stress, it is possible that different varieties may employ alternative strategies when faced with similar stressors. Studying the stress response using multiple functional traits may reveal important clues about complex adaptive strategies plants employ to cope with saline stress induced by climate change.

## Plant Materials and Methods

*Experimental design*. To examine variation in response to saline stress between cultivated and wild varieties of purslane, two isogenic varieties of purslane (T-16 and WI-9) were grown in two saline treatments (0 or 200 mM NaCl). T-16 was derived from the seeds of single plant of the cultivated variety ‘Tall Green’ (obtained from Wild Garden Seed, www.wildgardenseed.com in 2009). WI-9 is a wild variety derived from seeds of a single plant collected in Madison, Wisconsin in 2009. Both varieties were maintained locally via self-fertilization for several generations. Twenty plants (replicates) of each variety were assigned to each of the two saline treatments (the actual number of plants in each analysis varies and is slightly less than 20 due to sample loss).

*Seedling establishment, saline treatments, and harvest*. Five to 10 seeds of *P. oleracea* were sown on the surface of MetroMix 360 potting soil in 3.5 inch diameter plastic pots. The pots were placed in an insect-free greenhouse, and their position randomized with respect to their variety and assigned treatment. Ample water was provided to all pots during the two-week seedling establishment period. At the end of the establishment period, plants were thinned to one plant per pot before saline treatments began. After the plants were thinned, the number of nodes on the main stem and the number of branches from the main stem were recorded, to verify that no significant pre-treatment differences existed among plants of the same variety assigned to the two treatments. We applied one of two treatments to each pot: 0 mM NaCl or 200 mM NaCl. The 0 mM NaCl pots were watered three times per week with 56 mL tap water. The 200 mM NaCl pots were watered three times per week with 56 mL of a 200 mM NaCl solution prepared in tap water. These saline treatments were chosen based on results from preliminary experiments (data not shown).

*Harvest and collection of morphological trait data*. After 10 treatments over 21 days (35 days from sowing), we recorded the number of nodes on the main stem, the number of axillary branches produced on the main stem, the number of leaves dropped from the main stem, and the number of flower structures produced by each plant. Several leaves, which emerged during the treatment period, and a small segment of stem were harvested for chemical analysis. The above-ground biomass was determined by cutting the stem at soil level, and drying the plant material in an 80°C oven for 3–5 days. All masses were determined to 0.1 mg on an analytical balance.

*Chemical analysis*. Proline levels were measured using the method of Bates *et al.*. [37] Briefly, pieces of fresh leaves from each plant were sliced into small pieces and placed in 3% sulfosalicylic acid solution, then shaken for 60 s in a Bead Beater (no beads were added). The supernatant was mixed with ninhydrin solution and glacial acetic acid, then heated at 100°C for 1 h. After extraction into toluene, the absorbance at 520 nm was recorded. Comparison was made to a calibration curve prepared with pure proline. Results are reported in *μ*g proline/g fresh leaf.

Betalain pigment levels were measured using the method of Kugler *et al.*. [38] Briefly, pieces of plant tissue were placed in 60% methanol along with two steel beads and shaken for 60 s in a Bead Beater. We recorded the absorbance of the supernatant across the visible spectrum using an Ocean Optics spectrometer. The indices developed by Elbe [39] were used to compute the amounts of each pigment. Results are reported in *μ*g pigment/g fresh tissue.

Total phenolic compounds were measured using the bicinchoninic acid (BCA) assay. [40] Briefly, pieces of fresh leaves were placed in 60% methanol along with two steel beads and shaken for 60 s in a Bead Beater. The supernatant was mixed with ammonium acetate buffer and Cu(II) solution. BCA solution was added. The absorbance at 558 nm was recorded and compared to a calibration curve prepared with gallic acid. Results are reported in *μ*g GAE/g fresh leaf (GAE = gallic acid equivalents).

*Statistics* Statistical analysis was carried out using R 3.3 [41] and the R package HandyStuff was used to draw the reaction norms. [42] Prior to conducting ANOVA, variables were examined using a normal probability plot (QQ plot) to assess whether the variables appeared to arise from a normal distribution. While some variables had outliers, no non-normal trend was evident and the data were not transformed. A result was considered significant if the p-value was ≤ 0.05.

## Results

**Fig 1:**
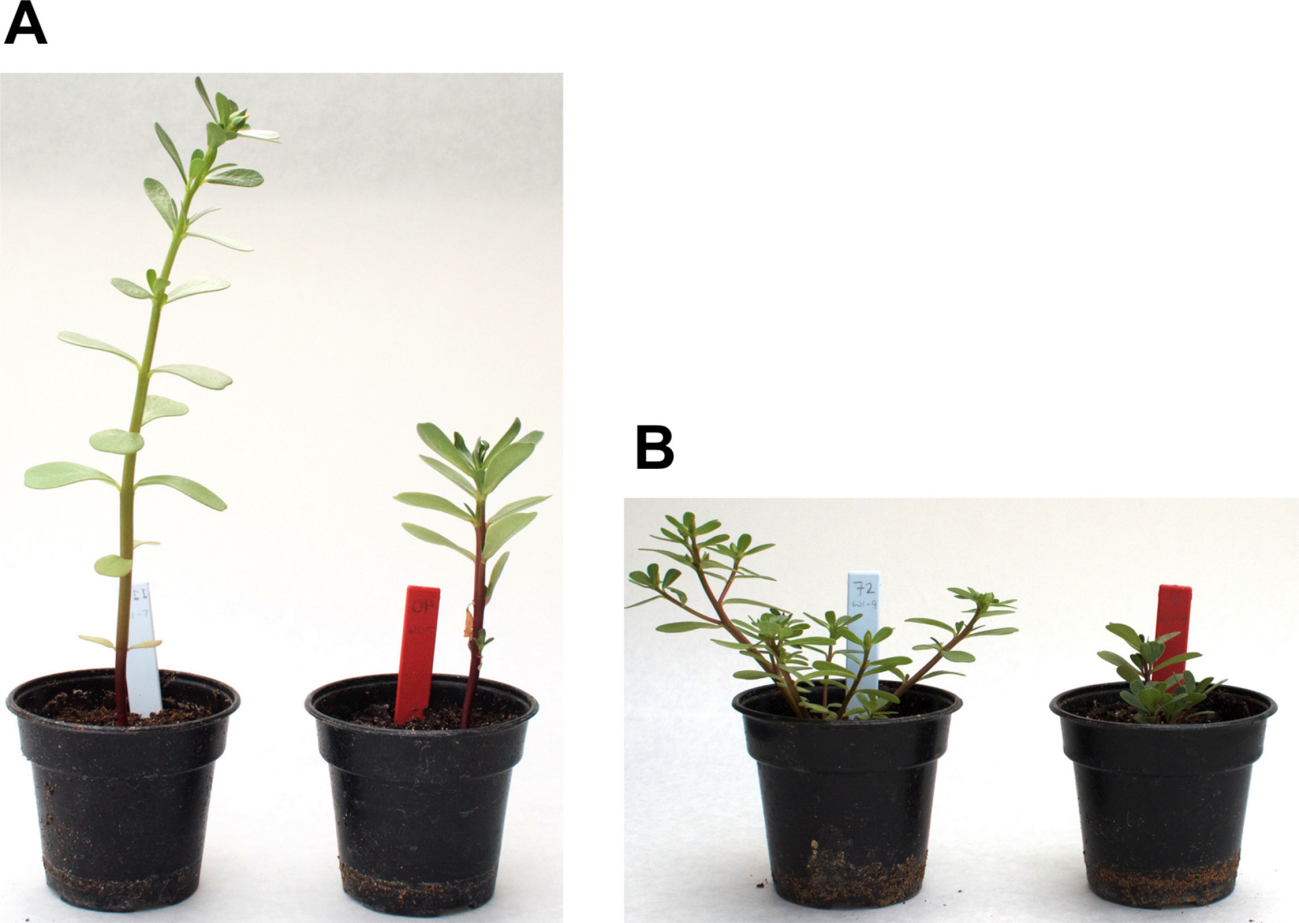
A: Variety T-16; B: Variety WI-9. Plants on the left were treated with 0 mM NaCl while plants on the right were treated with 200 mM NaCl.

Both varieties of purslane responded to saline stress with significant changes in morphological and physiological traits including a reduced investment in reproductive structures (Fig 1). However, the two varieties varied significantly in their specific responses to the saline environment, demonstrating that these varieties of *Portulaca oleracea* employ alternative strategies to cope with this type of stress.

### Morphological Traits

**Fig 2:**
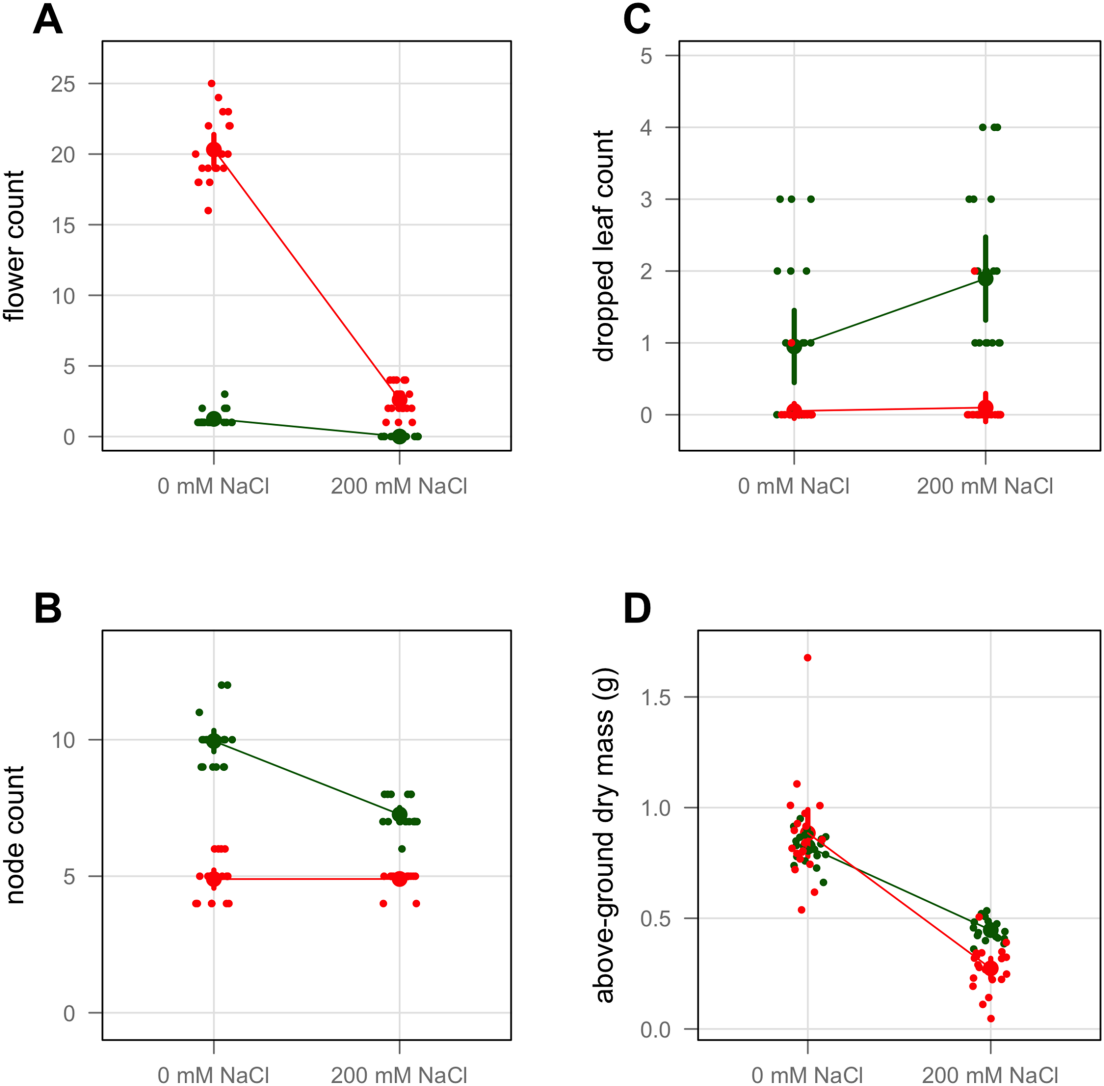
Morphological traits at 21 days. Variety T-16 shown in green. Variety WI-9 shown in red. Large point is the mean of the category. Small points are the sample values, jittered horizontally. Error bars correspond to the 95% confidence interval.

At the end of the 14-day establishment period, ANOVA was used to verify that the seedlings assigned to each treatment were similar within a variety, prior to the actual start of treatments (data not shown). Fig 2 shows the variation in morphological traits with saline stress after 21 days of treatment. There were significant variety by treatment interactions for all variables (Table 1). Both varieties produced fewer flowers and less dry biomass under saline stress. The decrease in flowering structures was particularly dramatic for variety WI-9. Variety T-16 produced fewer nodes and dropped more leaves from the main stem under the saline treatment than variety WI-9. However, variety WI-9 showed little change for these two variables under saline stress (see also S1, S2 and S3 Figs).

**Table 1:** ANOVA results for morphological traits.

**trait**	**term**	**df**	**SS**	**MS**	**F-statistic**	**p-value**
**flowers**	treatment	1	1652.3	1652.3	950.4	5.70E-44
	variety	1	2286.9	2286.9	1315.4	7.16E-49
	treatment:variety	1	1320.9	1320.9	759.7	1.17E-40
	Residuals	74	128.7	1.7		
**nodes**	treatment	1	40.2	40.2	92.3	1.19E-14
	variety	1	268.1	268.1	615.7	1.32E-37
	treatment:variety	1	35.3	35.3	81.1	1.63E-13
	Residuals	74	32.2	0.4		
**dropped leaves**	treatment	1	4.2	4.2	5.2	2.59E-02
	variety	1	35.3	35.3	43.9	4.83E-09
	treatment:variety	1	3.9	3.9	4.9	3.03E-02
	Residuals	74	59.5	0.8		
**dry mass**	treatment	1	4.8	4.8	265.1	3.61E-26
	variety	1	0.1	0.1	3.4	6.99E-02
	treatment:variety	1	0.3	0.3	14.7	2.63E-04
	Residuals	74	1.3	0.0		

### Physiological Traits

**Fig 3:**
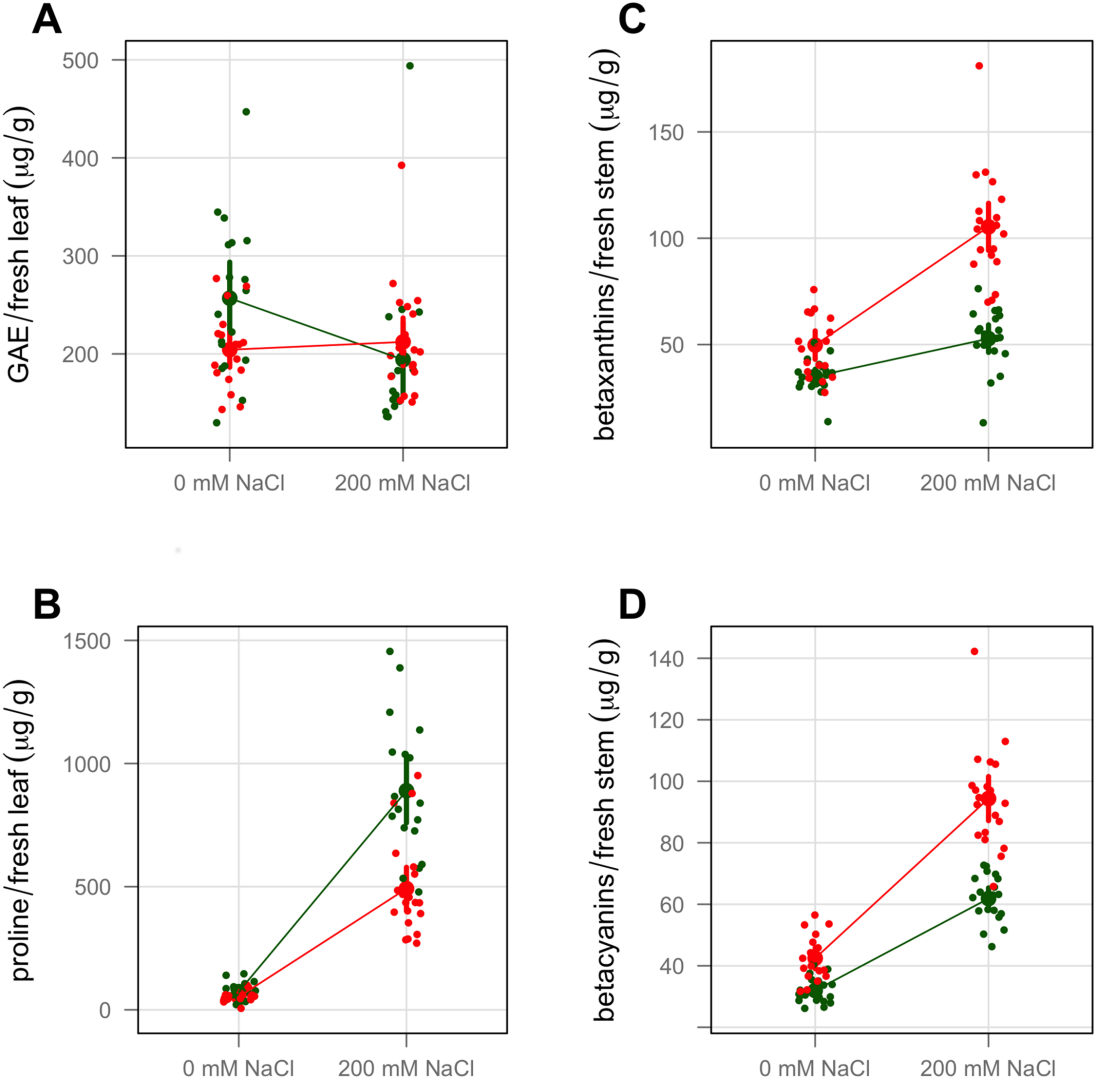
Physiological traits at 21 days. Variety T-16 shown in green. Variety WI-9 shown in red. Large point is the mean of the category. Small points are the sample values, jittered horizontally. Error bars correspond to the 95% confidence interval. A: total phenols. GAE = gallic acid equivalents. B: proline. C: stem betaxanthins. D: stem betacyanins.

The BCA assay was conducted as a measure of the total concentration of phenolic compounds which can serve as antioxidants. The results indicated a modest variety by treatment interaction, but no statistically significant main effects (Fig 3 Panel A and Table 2). The total phenols (measured as GAE or gallic acid equivalents) of variety T-16 decreased about 25% with increased salinity, but variety WI-9 remained largely unchanged. The concentration of the amino acid proline after 21 days of treatment exhibited a significant variety by treatment interaction (Fig 3 Panel B and Table 2). Variety T-16 responded to the saline stress by producing about 75% more proline than variety WI-9, per gram of fresh mass.

**Table 2:** ANOVA results for physiological traits.

**trait**	**term**	**df**	**SS**	**MS**	**F-statistic**	**p-value**
**phenols**	treatment	1	13376.4	13376.4	3.0	8.57E-02
	variety	1	4928.3	4928.3	1.1	2.94E-01
	treatment:variety	1	23082.1	23082.1	5.2	2.51E-02
	Residuals	70	308182.7	4402.6		
**proline**	treatment	1	7244272.3	7244272.3	244.6	7.44E-25
	variety	1	829534.0	829534.0	28.0	1.25E-06
	treatment:variety	1	672424.0	672424.0	22.7	9.54E-06
	Residuals	72	2132398.0	29616.6		
**stem betaxanthins**	treatment	1	27421.9	27421.9	97.4	4.42E-15
	variety	1	22083.2	22083.2	78.5	3.44E-13
	treatment:variety	1	6880.5	6880.5	24.4	4.73E-06
	Residuals	73	20545.7	281.4		
**stem betacyanins**	treatment	1	33310.0	33310.0	329.1	9.25E-29
	variety	1	9019.3	9019.3	89.1	2.81E-14
	treatment:variety	1	2327.0	2327.0	23.0	8.37E-06
	Residuals	73	7389.8	101.2		

Betalain pigment concentrations were measured in the stem tissue. Fig 3 Panels C and D shows the effect of salinity on the production of betaxanthin (yellow) pigments and betacyanin (purple-red) pigments. These pigments are produced in a layer just a few cells thick in the cortical tissue under the stem epidermis, but the extraction process includes all stem tissue (S1 Photo). As a result, the values reported here are low; the concentration of pigment is diluted by a large amount of interior tissue that does not contain betalain pigments. Even so, there was a significant variety by treatment interaction for both pigment classes. Variety WI-9 increased production of betaxanthin and betacyanin pigments under saline stress more than variety T-16. This result corresponds to our visual observations: WI-9 stems were distinctly more red-purple than T-16 stems, especially so in the saline treatments. Our analysis of betaxanthin and betacyanin pigments in the leaves revealed that both varieties increased pigment production with saline stress, but there was no variety by treatment interaction in the leaves (S4 and S5 Figs).

### Saline Stress Reveals Alternative Strategies

**Fig 4:**
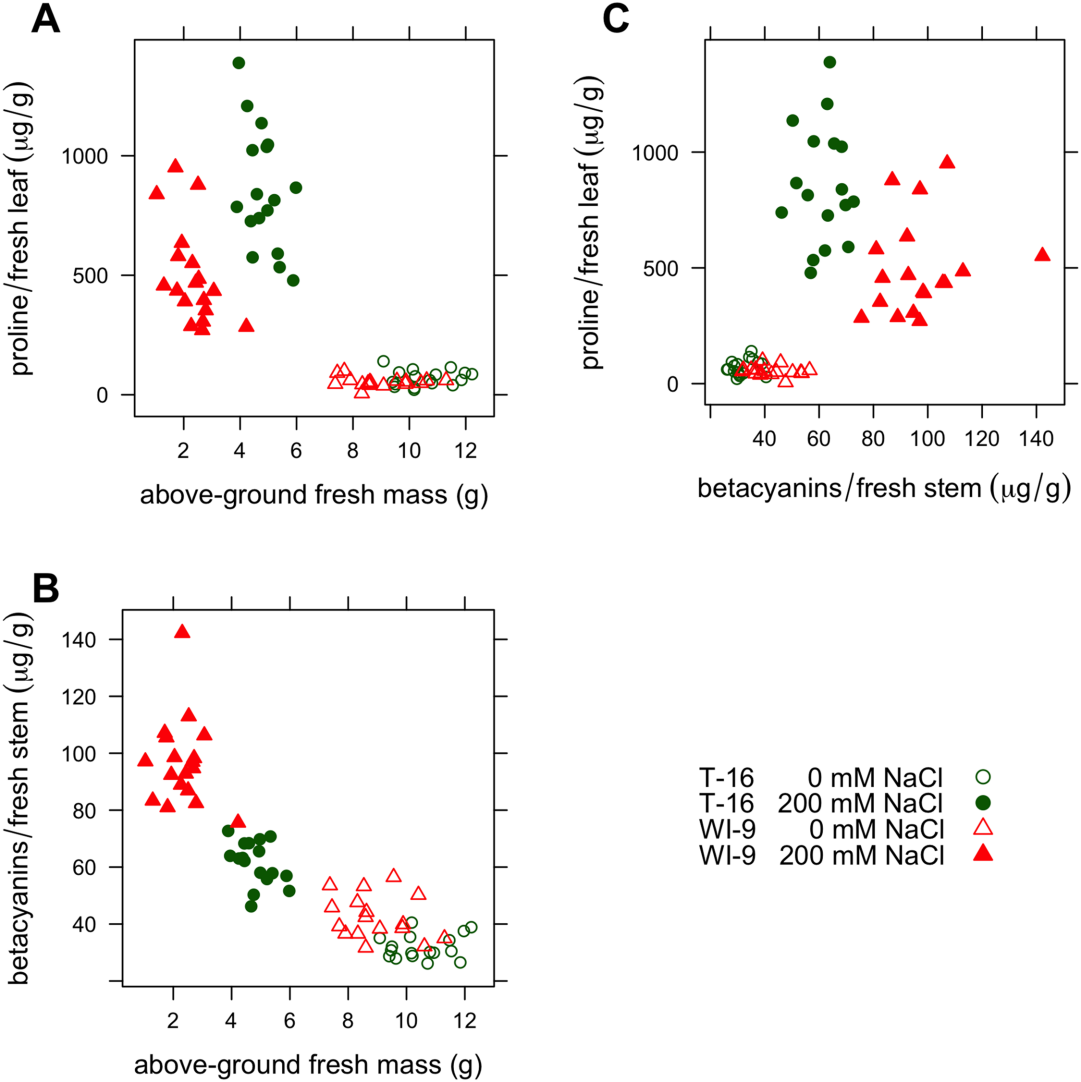
Relationships among selected variables.

Examining pairwise combinations of variables reveals that the two purslane varieties employ alternative strategies when faced with saline stress (Fig 4). The above-ground fresh mass is a surrogate measure for reproductive fitness. In the absence of saline stress, variety WI-9 produces slightly smaller plants on average, but there is no significant variation between the two varieties. In addition, both varieties produce the same amount of proline (Fig 4, panel A). However, when exposed to the 200 mM NaCl treatment, variety T-16 loses less mass but invests more in the production of proline, compared to WI-9 plants. Comparing the above-ground fresh mass with the concentrations of betacyanin pigments in the stems, one sees an opposite trend (Panel B). In this case, variety WI-9 exposed to 200 mM NaCl invests more in the synthesis of betacyanins in the stem, compared to the T-16 plants. These alternative strategies (investments) are even more apparent in Panel C, which compares the concentration of proline directly to the stem betacyanin concentration. In this case, both varieties are very similar in the absence of NaCl, but under the 200 mM NaCl treatment, they diverge substantially. Plants of variety T-16 investing relatively more in proline and variety WI-9 invests relatively more in betacyanins. While both proline and betalain pigments undoubtedly play multiple roles, one interpretation of these data is that variety T-16 employs proline as a primary antioxidant to protect against stress while variety WI-9 employs betacyanins in this role.

**Fig 5:**
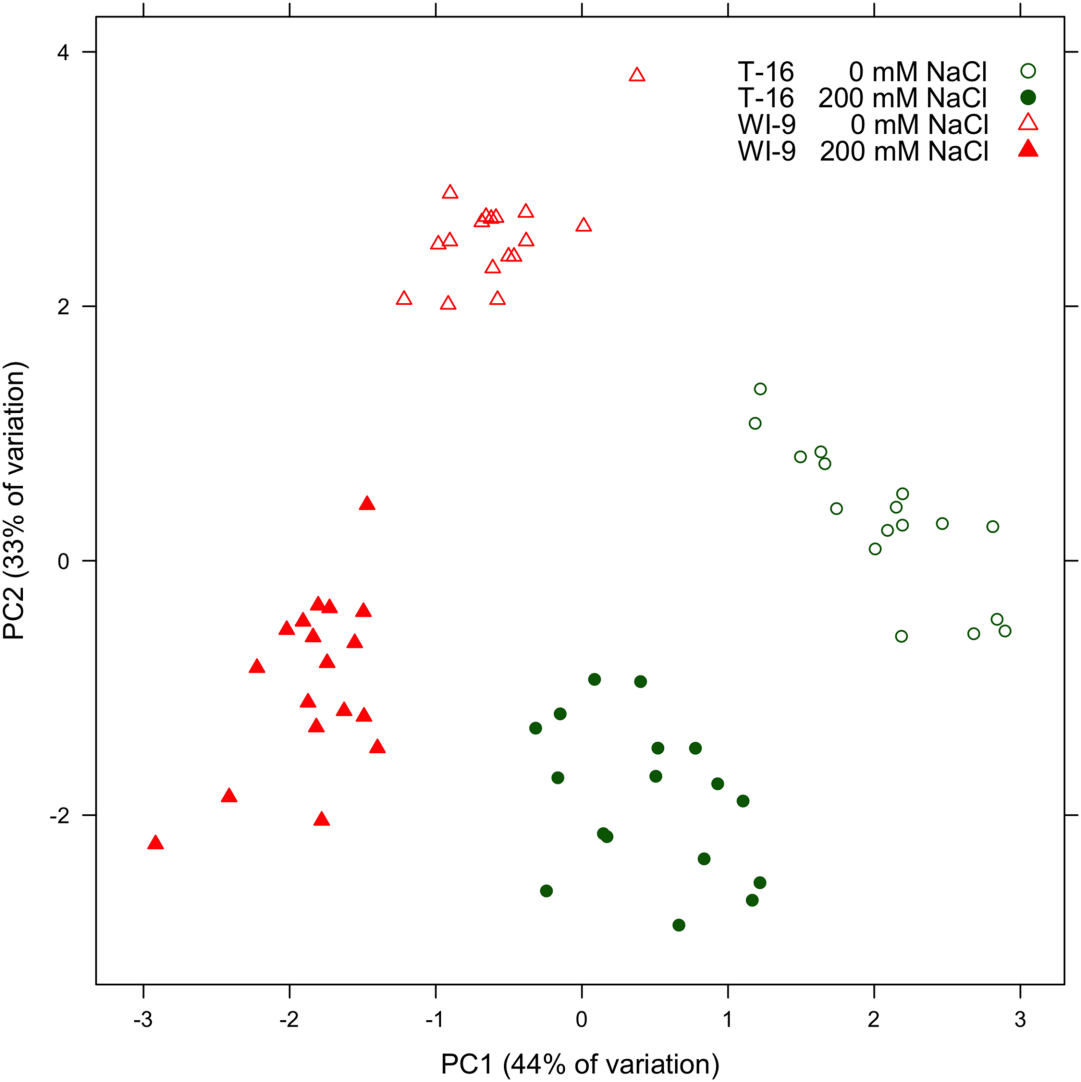
Score plot.

The alternative strategies for responding to saline stress are also evident in a principal components analysis (PCA) that considers several variables simultaneously (Fig 5). Variables included were node count, flower count, branch count, dropped leaf count, above-ground dry mass, proline concentration, and concentration of betacyanins in the stem. The inclusion of additional variables, such as the stem betaxanthin concentrations, gave poorer results, suggesting that the chosen variables best represented the underlying biology and chemistry. Values were scaled to unit variance prior to PCA. In this plot, excellent separation is observed according to the variety by treament categories. Principal component 1 largely reflects the separation by variety, while principal component 2 largely reflects the separation by treatment.

## Discussion

In this study, we have investigated morphological and physiological traits which characterize the response of purslane to saline stress. Morphological traits, such as the number of reproductive structures and above-ground biomass, serve as proxies for reproductive fitness. Physiological variables, on the other hand, provide a snapshot of the biochemical changes invoked by the stressor. We have described substantially different responses to saline stress in the two varieties of purslane that we studied. Such variety by treatment interaction effects are due in part to genetic differences between the varieties, and in part to phenotypic plasticity, the ability of a variety to respond in a flexible way to different environmental conditions. Phenotypic plasticity may reveal genetic variation which is hidden when the environmental conditions are relatively benign. However, under appropriate stress, this variation can be uncovered when a particular variety performs better under stress. Phenotypic plasticity is one of the factors that can drive speciation [43] and can serve as a buffer against extinction in the face of climate change. [35, 44] Studying the relationships among plastic responses of multiple traits yields a holistic view of purslane’s available strategies when responding to saline stress.

In general, plants react to salinity stress using some combination of three cellular mechanisms: excluding Na^+^ from the tissues (by preventing it from entering the roots or sequestering it in vacuoles), adapting as much as possible to the toxicity of excess Na^+^ in the tissues, and by adjusting to the increased osmotic pressure (which is more generic and can be induced by many molecular species). Complicating things, the response to Na^+^ is intertwined with the transport and balance of water, K^+^ and Cl^-^ in a poorly understood manner. [45] There is also a temporal aspect to the response as described by Munns. [46] The response of purslane to salt stress which we observed appears to involve the ionic phase identified by Munns in which the concentration of Na^+^ in the plant cells become toxic. This phase occurs after a shorter acclimation to the osmotic insult, and is characterized by the death of older leaves (dropped leaves in this study) and greatly reduced growth of new tissues (above-ground fresh mass in this study). At the concentrations and time frame employed in this study, purslane is unable to exclude NaCl from its tissues, consistent with the work of Teixeira and Carvalho [47] who found that Na^+^ concentrations in the tissues increased with increasing salinity of the water, using salinity ranges similar to this work.

Other researchers have reported studies of purslane under saline stress, measuring a variety of traits. [47–50] However, only a limited number of studies considered the traits explored here. These studies will be discussed in the next few sections.

### Morphological Traits

The morphological traits measured in this study serve as proxies for reproductive fitness, as they represents investments in reproductive structures and the tissues that support them. [51] In our experiments, the flower count, node count, and above-ground dry mass all decreased with increasing salt concentration, and the dropped leaf count increased (Fig 2). Both varieties showed substantial, statistically significant reduction in these fitness proxies under increased salinity, though the extent of the response varied between the two varieties (Table 1). These results demonstrate that 200 mM NaCl severely stresses purslane without killing it, and suggest that purslane is not strictly a glycophyte nor is it a halophyte (though Al Nasir *et al.* consider purslane to be a halophyte [52]). The other morphological traits we measured, namely the branch count, the above-ground fresh mass and the ratio of above-ground dry to fresh mass (S1, S2 and S3 Figs) also demonstrated a significant stress response for both varieties, as well as substantial differences between the varieties. These observations are consistent with the overall habits and architectures of the two varieties (Fig 1). Variety T-16 (domesticated ‘tall-green’) grows tall, produces many leaves, flowers late, branches late and flowers largely at the apical meristem. On the other hand, variety WI-9 (wild-type) sprawls, branches frequently and flowers early at most leaf axils. These differences drive some of the variation observed, but the overall trends we observe also reflect the suppression of flowering in WI-9 and node formation in T-16 by the saline treatment. Our measures of biomass are consistent with those of Alam *et al.* who have studied a number of purslane accessions with regard to salinity tolerance. [53–55]. They also found that the loss of biomass depended upon the variety, and in general, was quite substantial. Similar trends were found for the number of flowers, leaves and dropped leaves, though the methodological details differed from our study.

### Physiological Traits

Phenolic compounds such as phenolic acids, anthocyanins (not found in purslane), flavonoids and tannins serve multiple roles in plants, including functioning as antioxidants. We assayed the total phenolic content of purslane using the BCA assay. [40] This assay responds to phenolic substances in a similar fashion as the more commonly used Folin-Ciocalteu assay [56] but is more sensitive, and the color development is stable. We observed a significant variety by treatment interaction in which the phenolic content of variety WI-9 was nearly inert to increased NaCl, while variety T-16 *decreased* the production under saline treatment (Fig 3 Panel A, Table 2). The values found here with the BCA assay are in the range of those found by Alam *et al.* for purslane using the Folin-Ciocalteu assay (after correction to a dry mass basis); they also observed that varieties may produce more *or* less total phenols under saline stress. [55]

Up-regulation of the amino acid proline in stressed plants has been known for decades (reviewed in [57]; see [58] for recent studies) and its role has generally been understood as an osmoprotectant, one of several small molecules that plants produce for this purpose. However, the question of ‘why proline?’ has been clarified with the observation that proline functions as an antioxidant via its secondary amine (absent from all the other amino acids). [59] Proline also plays other intertwined and somewhat poorly understood roles. [60] In our experiment, each variety had similar proline concentrations in the absence of saline stress, but dramatically increased concentrations when watered with 200 mM NaCl. Variety T-16 produces more proline on average (Fig 3 Panel B). In a study of purslane on saline soils in Jordan, Al Nasir *et al.* found that the amount of proline increased under stress in autumn but decreased in the spring. [52] On the other hand, Yazici *et al.* [50] and Kafi and Rahimi [61] found significant increases in proline in experiments very similar to the one described here.

The role of betalain pigments in plant physiology is less well-studied than that of the anthocyanins, but presumably the two pigment families fulfill similar functions. Like proline, the betalain pigments are antioxidants. [62, 63] We observed significant variety by treatment interactions for both stem betacyanins and stem betaxanthins (Fig 3 Panels C and D). This quantitative analysis is in line with our visual observations: the saline stressed plants are strikingly more red-purple.

These two pigment categories correlate strongly (S6 Fig). This likely occurs because they share a common biosynthetic intermediate, betalamic acid, and the downstream reactions are spontaneous, not enzyme catalyzed. [64] Thus any betalamic acid produced can react with any available amino acids to give betaxanthins, or with a cyclo-DOPA derivative to give betacyanins, with more or less equal likelihood.

To our knowledge, this is one of the few studies to quantify an increase in betalain pigments with saline stress. Chang-Quan *et al.*, investigating *Suaeda salsa*, a C-3 halophyte, observed that betalain levels decreased initially under saline stress but then rebounded, and never exceeded their initial levels. [65] Jain and Gould reported that red, but not green, morphs of the coastal succulent *Disphyma australe* increase their betalain content 10-fold in response to salinity. [66] The only report on *P. oleracea* is from Alam *et al.*, who briefly noted a visual increase in red-pink pigments with saline stress. [53]

### Alternative Investments as Adaptive Strategies

Considering the responses we observed as a whole, it is clear that the two varieties of purslane that we studied adopt different strategies when investing in resources to cope with saline stress. These alternative responses consist of complex, simultaneous changes in multiple traits, and may represent evidence of a trade-off among multiple alternatives for coping with saline stress. The most noteworthy changes in traits due to saline stress within the two varieties are summarized in Table 3.

**Table 3:** Summary of noteworthy changes in traits under saline stress.

**trait**	**variety T-16**	**variety WI-9**
flower count	∼	--
node count	-	∼
dropped leaves	+	∼
dry mass	-	--
total phenols	-	∼
proline	++	+
stem betacyanins	+	++

Key: ∼ largely unchanged; - decreased; -- strongly decreased; + increased; ++ strongly increased.

Strategies for the allocation of metabolic resources can be better appreciated if the data are examined holistically instead of considering single traits in isolation. Overall, variety WI-9 suffers a substantial reduction in flowering and above-ground dry biomass under saline stress, and responds with a large increase in stem betacyanin pigments and a modest increase in proline concentration. Variety T-16 on the other hand suffers modest decreases in above-ground biomass and node count, an increase in the number of dropped leaves, greatly increased proline and only modestly increased stem betacyanins. With respect to proline and stem betacyanins, variety T-16 inverts the response of variety WI-9. This is most apparent in panel C of Fig 4 and this plot is probably the best illustration of the different strategies employed by these two varieties. These alternative responses are clearly reflected in the PCA score plot, where one sees a wide separation for each variety by treatment combination (Fig 5). This is further evidence that each variety is allocating its morphological and physiological resources in the face of stress according to a different strategy. This may be evidence for a tradeoff between alternatives; perhaps allocation to betalains precludes up-regulation of proline under severe saline stress. If the experiment were carried out longer, some of these trends might be even more apparent (for instance, at 21 days, T-16 has barely begun to flower).

Proline, betalain pigments and phenols are metabolites with multiple roles. Proline is an osmoprotectant, antioxidant and serves other roles as well. [60] Betalains function to attract pollinators, to protect tissues from UV light, and as antioxidants. Phenolic compounds include many subtypes, each with their own, often poorly known functions, and also possess antioxidant behavior to varying degrees. [67] Salinity induces responses similar to drought since it reduces the water available to the tissues, and both types of abiotic stress are known to increase reactive oxygen species (ROS) which are critically involved in signalling pathways within plants. [68] Thus ROS are damaging to plant tissues and their concentration must be limited, but they are also necessary as secondary messengers, and complete suppression would be as problematic as too much production. [69] It was recently reported that *Arabidopsis* responds rapidly to saline stress with a Ca^2+^ signaling wave which induces ROS related genes. [70] In our experiment, we found that some antioxidants increased (proline, betalains) but the total phenols followed a more complex pattern. Looking at the activity of enzymes that quench ROS, Yacizi *et al.*, using 2-month old purslane plants, also found a complex pattern of increases and decreases over time. [50] In that study, the levels of proline steadily increased with time and NaCl concentration, but the concentration of malondialdehyde, a measure of membrane damage, showed a delayed response, only increasing at 30 days of treatment and not at 18 days. Coupled with our results, the observed patterns almost certainly reflect that plants need to maintain a balance and avoid a too ‘heavy-handed’ antioxidant response by decreasing some antioxidant species while increasing others. Türkan and Demiral have noted the need for balance between antioxidant quenching enzymes and small molecule antioxidants in various cellular compartments, as well as the partially overlapping and redundant nature of the system. [71] It also reflects the heterogeneity of the ‘total phenols’, as they are really many compounds which may have widely varying roles and individual concentrations which may increase or decrease under stress. Similarily, it is impossible to disentagle whether the increased proline concentration is functioning as an osmoprotectant or an antioxidant. Such an approach is too reductionistic, as proline is clearly capable of both functions. Betalains have not been identifed as osmoprotectants, but are proven antioxidants. Betacyanins are also phenolic substances, and presumably measured in the BCA assay. Since our control and experimental plants were exposed to the same light regime and the plants are self-pollinating, the pigment increase has nothing to do with screening UV light or attracting pollinators. Thus their antioxidant behavior is probably more relevant in this system.

In this work we have identified two varieties of purslane that employ alternative strategies in allocating resources in the face of saline stress. This establishes purslane as a suitable model system for the study of saline stress and the molecular basis for differential responses. We also report for the first time a significant increase in betalain pigments due to saline stress which occurs rapidly in young plants, particularly in the wild type WI-9. The phenotypic plasticity observed here must of course arise from slightly different genes, which respond differentially to environmental signals to give a better (or worse) adapted proteome for a given stress condition. [36] The proteome in a given situation represents a suite of enzymes and transcription factors which is ideally adapated to the environmental inputs, and effectively supervises the organism’s response to the stressor. This results in varying concentrations of the final metabolites, some of which we have studied in this work.

## Supporting Information

S1 Photo Cross section of T-16 stem showing the layer of pigment containing cells. (TIFF)

S1 Fig Branch count at 21 days. See the caption of Fig 2 for interpretation.
(TIFF)

S2 Fig Above ground fresh mass at 21 days. See the caption of Fig 2 for interpretation.
(TIFF)

S3 Fig Ratio of dry to fresh mass at 21 days. See the caption of Fig 2 for interpretation.
(TIFF)

S4 Fig Leaf betaxanthins at 21 days. See the caption of Fig 2 for interpretation.
(TIFF)

S5 Fig Leaf betacyanins at 21 days. See the caption of Fig 2 for interpretation.
(TIFF)

S6 Fig Comparison of stem betacyanins concentration to stem betaxanthins concentration. The correlation between these variables, ignoring group membership, is 0.93.
(TIFF)
