# Supplementary figures and images for: Alternative Strategies in Response to Saline Stress in Two Varieties of Portulaca oleracea (Purslane)
Source: PLoS One. 2015 Sep 23;10(9):e0138723. doi: 10.1371/journal.pone.0138723 (PMC4580602; doi:10.1371/journal.pone.0138723)

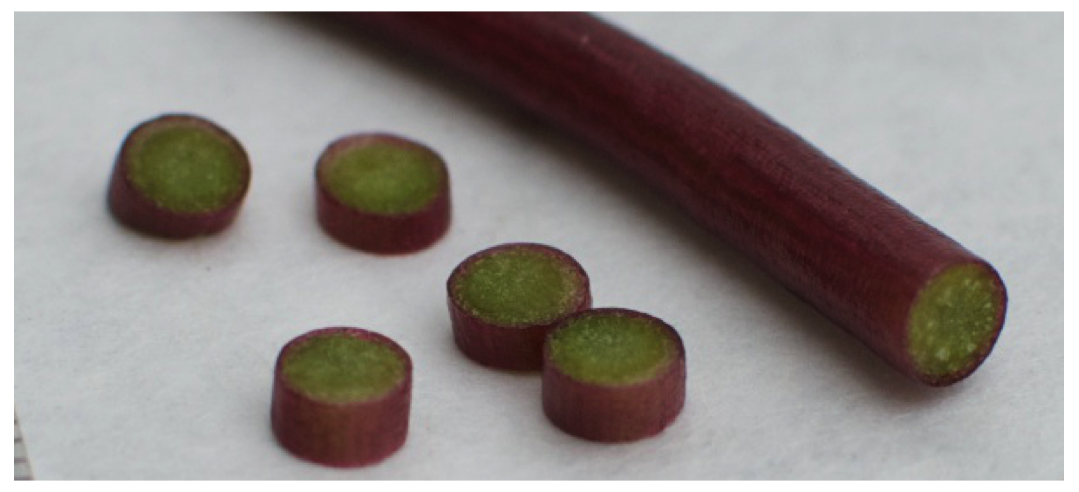

Supplement: S1 Photo — (TIFF) [file pone.0138723.s001.tiff]

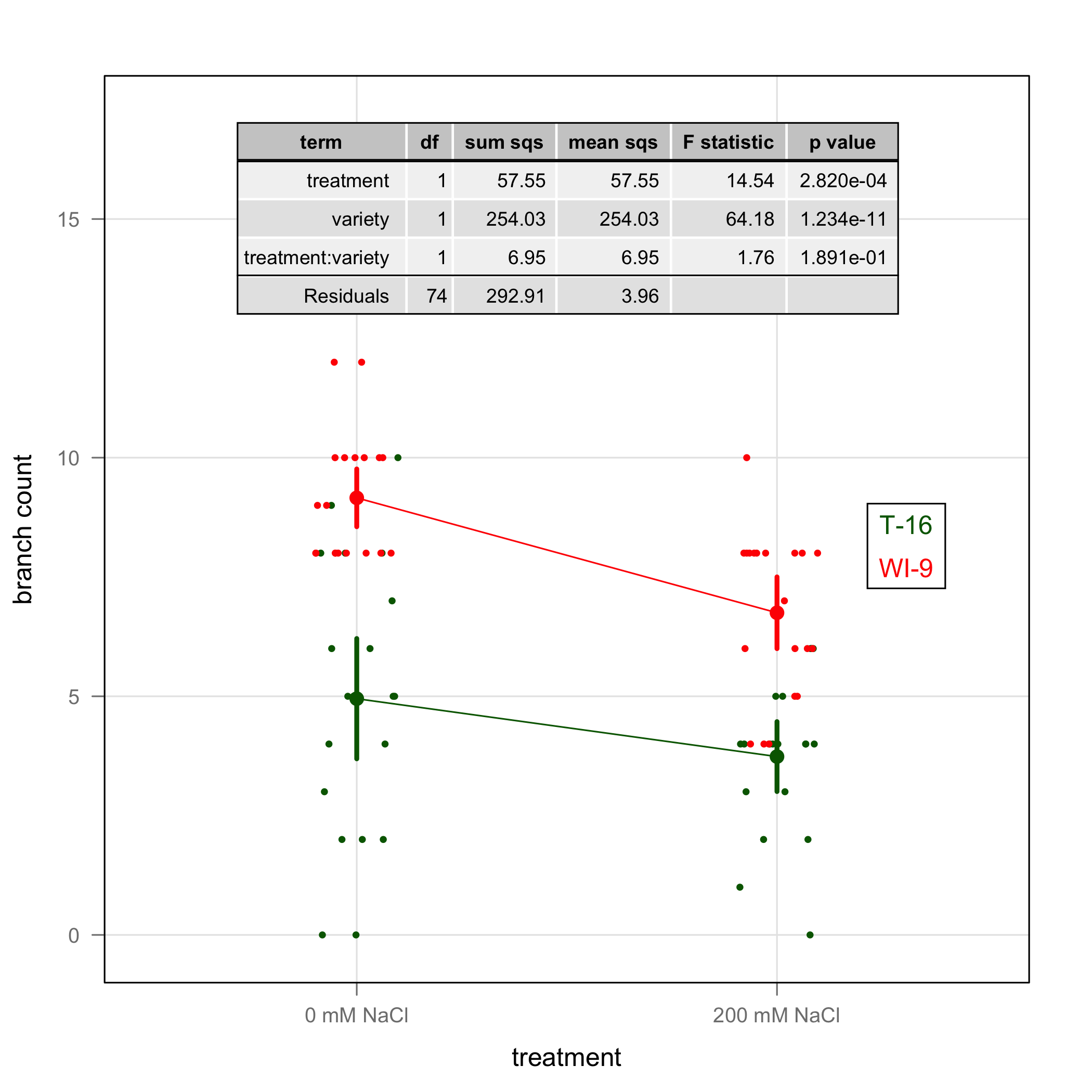

Supplement: S1 Fig — See the caption of Fig 2 for interpretation. (TIFF) [file pone.0138723.s002.tiff]

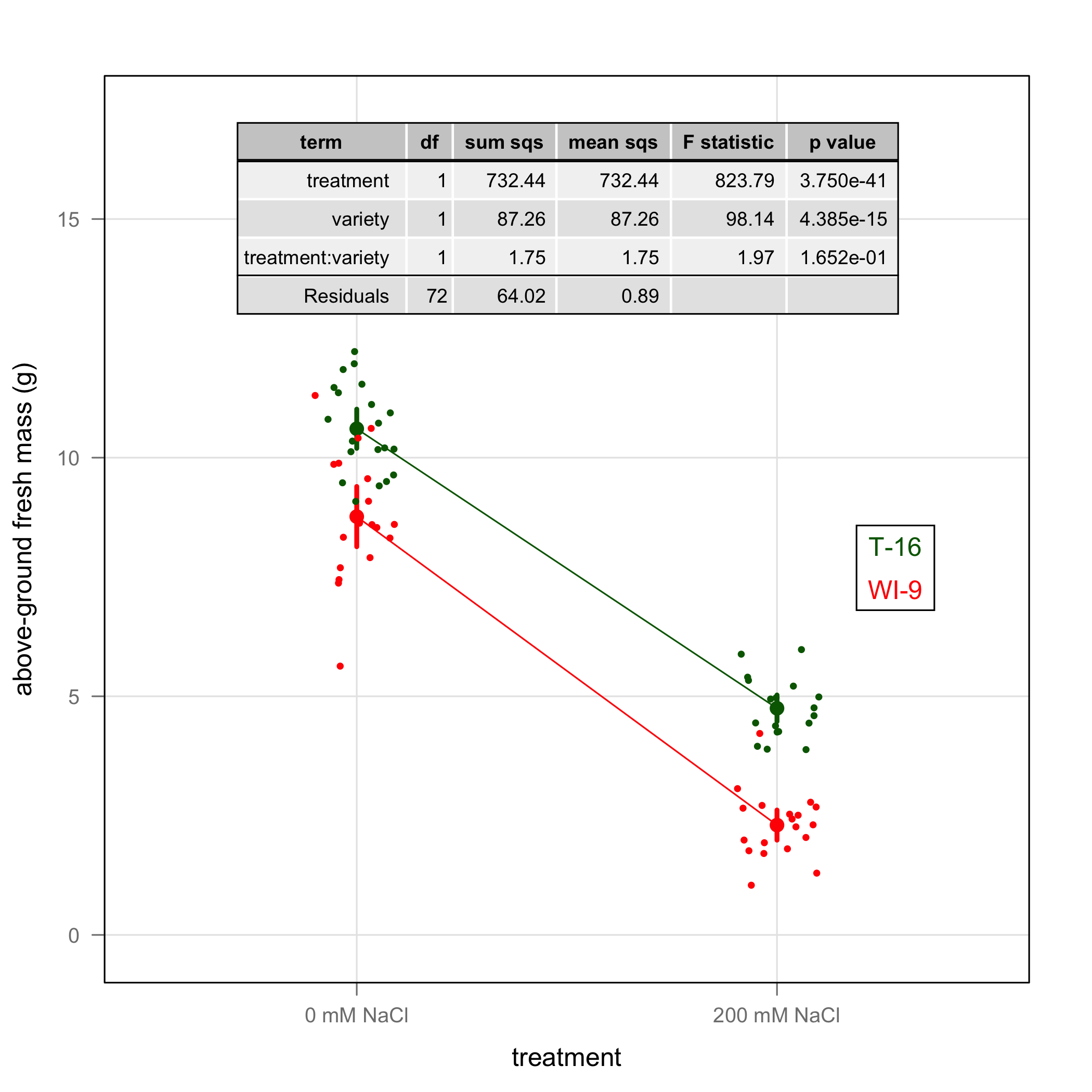

Supplement: S2 Fig — See the caption of Fig 2 for interpretation. (TIFF) [file pone.0138723.s003.tiff]

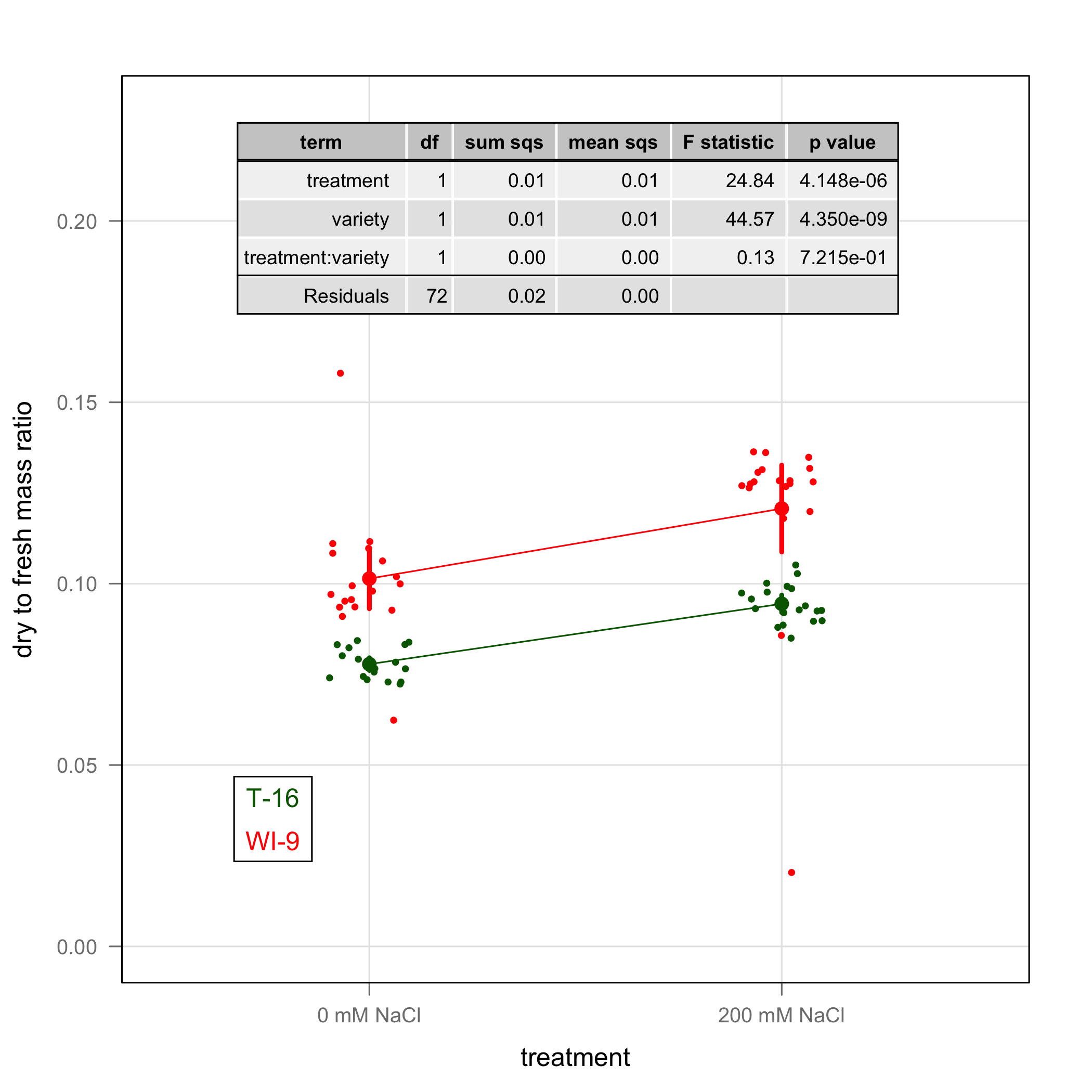

Supplement: S3 Fig — See the caption of Fig 2 for interpretation. (TIFF) [file pone.0138723.s004.tiff]

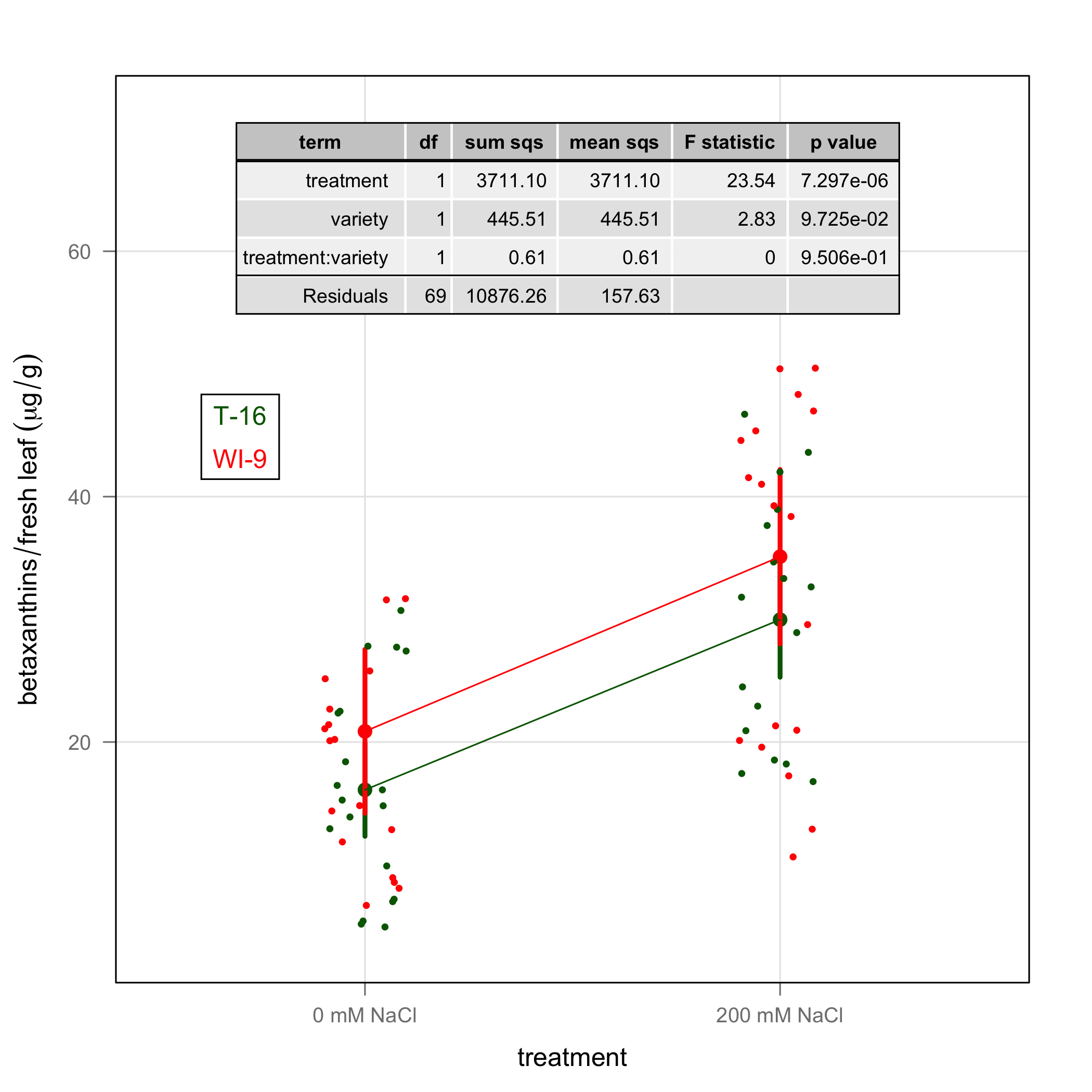

Supplement: S4 Fig — See the caption of Fig 2 for interpretation. (TIFF) [file pone.0138723.s005.tiff]

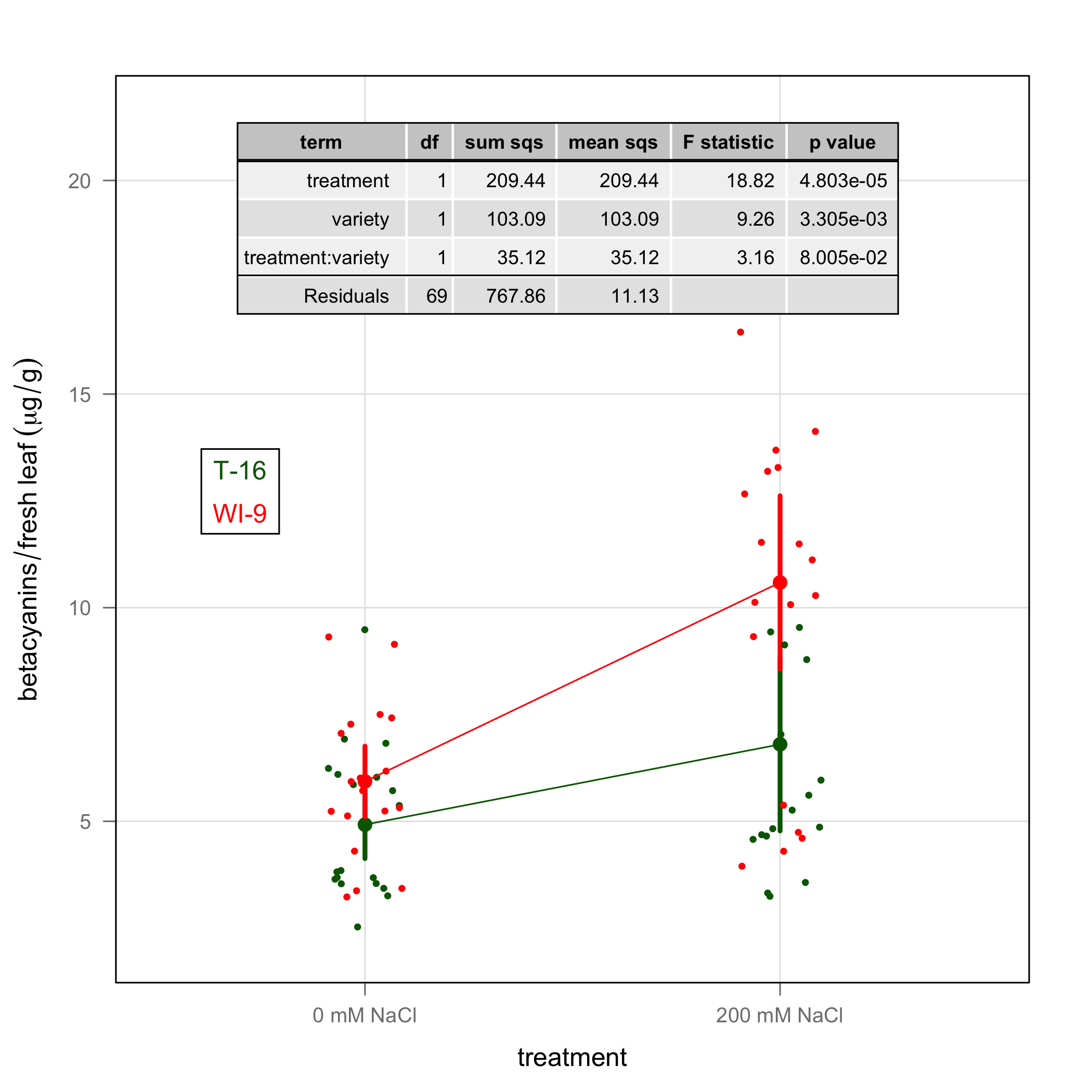

Supplement: S5 Fig — See the caption of Fig 2 for interpretation. (TIFF) [file pone.0138723.s006.tiff]

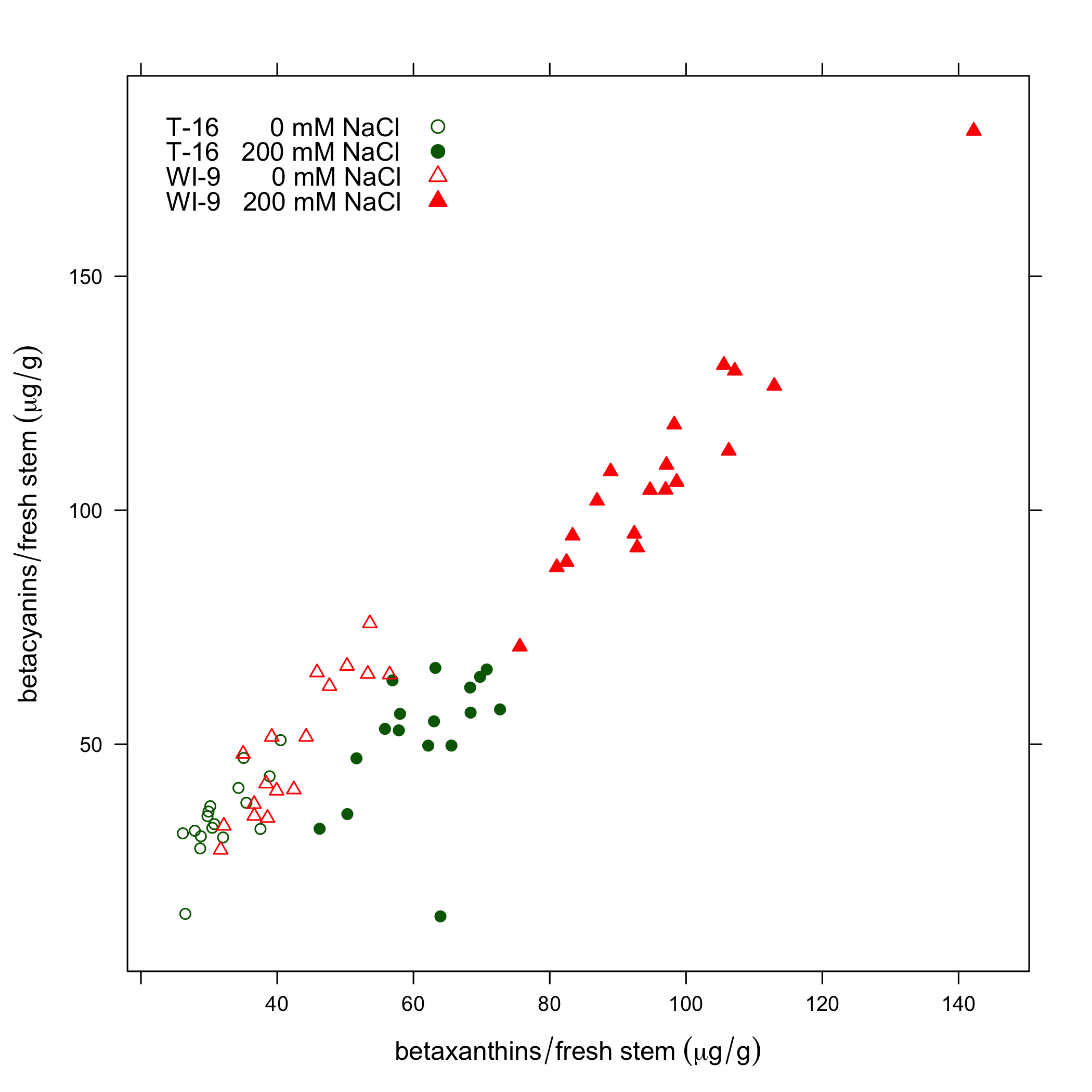

Supplement: S6 Fig — The correlation between these variables, ignoring group membership, is 0.93. (TIFF) [file pone.0138723.s007.tiff]
